# Self-test for HIV infection diagnosis: educational technology development and validity

**DOI:** 10.1590/0034-7167-2024-0627

**Published:** 2026-03-30

**Authors:** Ana Luiza Carsoni Alves de Almeida, Karyanna Alves de Alencar Rocha, Júlia Freitas Gomes, Marcela Antonini, Henrique Ciabotti Elias, Elucir Gir, Renata Karina Reis

**Affiliations:** I Universidade de São Paulo. Ribeirão Preto, São Paulo, Brazil

**Keywords:** Self-Testing, Diagnosis of HIV Infection, Educational Technology, Validation Study, Disease Prevention., Autoprueba, Diagnóstico de la Infección por el VIH, Tecnología Educativa, Estudio de Validación, Prevención de Enfermedades.

## Abstract

**Objectives:**

to develop and validate an educational booklet on HIV self-testing.

**Methods:**

the study was developed in four stages: literature review; booklet development; expert validity; and target audience assessment. The study was conducted between February and July 2024. The Content Validity Index was used for analysis, with values above 0.80 considered valid.

**Results:**

the 12-page booklet provides guidance on performing and interpreting HIV self-tests. It was validated by 13 experts and seven participants, achieving Content Validity Indexes of 0.92 and 0.95, respectively. The results indicate that the material exhibits content, appearance, and semantic validity.

**Conclusions:**

the booklet “Complete guide to performing the HIV self-test” has the potential to strengthen health literacy, supporting counseling and educational activities for the population in the correct use of self-test

## RESUMEN

**Objetivos:**

construir y validar un folleto educativo sobre la autoprueba para el diagnóstico del VIH.

**Métodos:**

el estudio se desarrolló en cuatro etapas: revisión bibliográfica; desarrollo del folleto; validación por expertos; y evaluación del público objetivo. El estudio se realizó entre febrero y julio de 2024. Se utilizó el Índice de Validez de Contenido para el análisis, considerándose válidos los valores superiores a 0,80.

**Resultados:**

el folleto de 12 páginas ofrece orientación sobre la realización e interpretación de las pruebas de autodiagnóstico del VIH. Fue validado por 13 expertos y siete participantes, alcanzando Índices de Validez de Contenido de 0,92 y 0,95, respectivamente. Los resultados indican que el material presenta validez de contenido, apariencia y semántica.

**Conclusiones:**

el folleto “Guía completa para la realización de la autoprueba del VIH” tiene el potencial de fortalecer la alfabetización en salud, apoyando actividades de consejería y educación para la población en la correcta realización de la autoprueba.

## INTRODUCTION

Human immunodeficiency virus (HIV) infection remains a significant public health challenge, even more than four decades after its identification. In 2023, there were 39 million people living with HIV worldwide, of whom 29.8 million were receiving antiretroviral therapy. Even so, 1.3 million new cases and 630,000 AIDS-related deaths were recorded globally. To address this reality, the Joint United Nations Programme on HIV/AIDS proposed an ambitious global target known as 95-95-95, which aims to diagnose 95% of people living with HIV, treat 95% of those diagnosed, and achieve viral suppression in 95% of those treated-a commitment to which Brazil is a signatory^(1)^. Countries that have made progress in reducing HIV incidence have invested in combination prevention strategies, addressing social, legal, and structural barriers such as stigma and gender inequality^(2)^. Among these strategies, expanding access to HIV testing is fundamental, and informing the population about testing locations is an essential action in this process^(3)^.

Among the promising strategies to expand HIV prevention and diagnosis, self-testing implementation stands out, recommended since 2016 by the World Health Organization and the Ministry of Health as part of combined prevention^(3)^. Therefore, expanding HIV testing is a global goal and a way to provide opportunities for HIV diagnosis. It constitutes the initial stage toward achieving the global objectives of managing the epidemic through diagnosis, treatment, and viral suppression. Self-testing offers advantages such as privacy, convenience, and accessibility. This strategy allows reaching populations with low testing rates, including those who have never been tested for HIV, contributing to meeting global diagnostic targets^(4-6)^.

In Brazil, self-testing is distributed free of charge by the Brazilian Health System (In Portuguese, *Sistema Único de Saúde* - SUS), which can be performed by the user using oral fluid or blood samples, promoting user autonomy. It is even offered in tele-consultations to expand its distribution and reach more vulnerable people who face difficulties in accessing the health system^(7-9)^. Furthermore, self-testing has been used as a strategy that contributes to combined HIV prevention, especially in partner selection based on test results, and is considered more effective than verbal reporting of sexual partners’ serological status^(10)^.

Therefore, implementing HIV self-testing in Primary Health Care is an effective tool for expanding access to diagnosis and overcoming barriers faced by population segments most exposed to social inequities and vulnerable groups. Due to its ease of use and convenience, it allows individuals to take the test at their preferred location, with results in 20 minutes^(8)^, promoting the empowerment of individuals in managing their health. Integrated with combined prevention initiatives, such as Pre-Exposure Prophylaxis, and strategic campaigns, self-testing facilitates timely diagnosis and represents another opportunity for regular HIV testing. However, to ensure its effectiveness, it is essential to provide appropriate guidance and counseling on kit use, beyond simply delivering the kit^(11-13)^.

Nurses play a fundamental role, together with the multidisciplinary team, in preventing HIV infection and other sexually transmitted infections (STIs) in the SUS Healthcare Network^(14,15)^, acting in the performance of pre and post-test reception and guidance of users who perform HIV self-testing, including referral to specialized services, risk management and treatment initiation in reactive cases^(16)^. Regular testing is an essential strategy for combating the epidemic. It is recommended that all sexually active people undergo HIV testing at least once a year and after any risky situation, such as unprotected sex, sharing needles, or sexual violence^(1-3)^. However, barriers such as stigma, discrimination, and difficulty in accessing healthcare services require increased dissemination of self-testing and clear guidelines for its appropriate use^(4,17,18)^.

Health literacy is essential for people to understand and use information about HIV prevention, testing, and treatment. Self-testing expands access to diagnosis, especially among vulnerable populations^(19)^. This study contributes to health education by developing and validating a booklet with clear and accessible language about self-testing using blood fluids. The booklet aims to reduce information barriers, promote autonomy, encourage regular testing, and strengthen educational initiatives, highlighting the central role of nurses in HIV prevention and achieving global health goals.

## OBJECTIVES

To create and validate an educational booklet content and appearance on self-testing for diagnosing HIV infection.

## METHODS

### Ethical aspects

The study was submitted to and approved by the Research Ethics Committee of the institution in charge, under Opinion 6,090,413, dated May 30, 2023. It followed the guidelines and regulatory standards for research involving human subjects in Brazil, as recommended by Resolution 466/12 of the Brazilian National Health Council and Circular Letter 02/2021, which established guidelines for procedures in research conducted in a virtual environment. All participants were informed about the objectives, risks, and benefits of this study, and data were collected after they agreed to and signed the Informed Consent Form (ICF), obtained through physical or digital documents.

### Study design, period and location

This methodological study was conducted in four stages: a narrative literature review; educational booklet development; validity by experts; and assessment by the target audience. The study was conducted at a Specialized Care Service (SCS) in a medium-sized municipality in the countryside of the state of São Paulo from December 2022 to July 2024. The research team included researchers, nurses, and graduate students working in nursing care for people with STIs.

### Development stages

The first stage of developing a booklet on HIV self-testing involved a narrative literature review, with an emphasis on the Ministry of Health’s recommendations on the use of self-tests using blood fluid obtained by fingerstick blood collection, made available by the SUS^(9)^. To illustrate self-testing stages along with the explanatory content, high-resolution photographs were produced by a professional in the clinical practice laboratory of the *Universidade de São Paulo, Escola de Enfermagem de Ribeirão Preto*, with the voluntary participation of a research group member. The images were captured in a suitable environment and used in infographics developed by a specialized designer, who was also responsible for the layout and visual presentation of the first version of the booklet. The selected content was organized in a step-by-step format in a digital file, with guidelines related to testing, prevention, and promoting autonomy in performing the test integrating the test description with health education content.

In the second stage, content and appearance validity were performed by a committee of experts. Expert identification and selection for this study were based on criteria that highlight experience and knowledge in healthcare, adapted from the Fehring Validity Model expert classification system^(20)^. These criteria include having recent clinical practical experience of at least one year in the area of interest and/or scientific knowledge, having completed graduate courses related to the study topic (master’s degree and/or doctoral degree) and having published in an indexed journal on the topic of interest.

Experts were selected through a search on the *Lattes* Platform and through snowball and convenience sampling techniques^(21)^, aiming to reach accessible participants from different regions of the country with proven experience and expertise, in accordance with inclusion criteria. An invitation was sent by email, containing study presentation, objectives, justification for participation, as well as a link to the ICF and the self-administered questionnaire on the Research Electronic Data Capture (REDCap^®^) platform.

The questionnaire consisted of two parts: the first gathered information about experts’ identification and their professional experience; the second contained instructions for completing the booklet and assessment criteria, totaling 35 questions. Of these, ten related to relevance and overall assessment (understanding of guidelines, motivation, and practical applicability); 17 addressed content and written language adequacy; and eight addressed aesthetic and visual quality, based on a questionnaire proposed in another study^(22)^. Participants had the option to provide suggestions and observations about the assessed attributes, such as adding relevant content and identifying the need for adaptation in accordance with scientific evidence.

In the third stage, educational booklet assessment with the target audience, the objective was to identify evidence of quality, adequacy for use, reliability, and relevance so that it could be used by a specific population segment. Thus, the booklet’s semantic and appearance were assessed with the target audience^(23)^, composed of users served at the Central Specialty Center, specifically at the SCS and Testing and Counseling Center of the municipality studied.

The study included literate adults aged 18 or older, HIV-positive or HIV-negative, and who had not previously self-tested for HIV. The inclusion of users with no prior self-testing experience aimed to assess language comprehension and the information provided in the booklet without biases related to prior knowledge. People with visual or cognitive impairments were excluded from the sample.

Participants were selected consecutively between December 2023 and February 2024. After recruitment and signing the ICF, data were collected individually in a private room, ensuring confidentiality, and recorded on REDCap^®^. Each participant received the printed booklet and answered a questionnaire with sociodemographic questions and 16 statements adapted from a previous study^(20)^, assessing content adequacy, relevance, clarity, and pertinence, including aspects of HIV self-testing and the necessary care for its performance.

### Data analysis

For data analysis, exploratory analysis was performed using descriptive statistics using measures of frequency, central tendency, and dispersion with the aim of describing and characterizing the profile of participants.

To analyze the agreement between experts and the target audience, the Content Validity Index (CVI) was used^(24,25)^, which measures the proportion of experts in agreement. Responses to each question were Likert-type with scores from 1 to 5: (1) totally disagree; (2) disagree; (3) neutral/indifferent; (4) agree; and (5) totally agree.

The CVI for each item was calculated using two metrics. The Item-Level Content Validity Index (I-CVI) was calculated by dividing the proportion of experts who considered an attribute relevant to a score of “5” or “4” or that required minor revisions by the total number of experts. Attributes that received a score of “1”, “2”, or “3” were revised. In this study, an acceptable CVI was considered to be 0.80 or greater, ensuring the validity and adequacy of the assessed attributes, as recommended by several authors in the field of content assessment^(23,24)^. The Global Content Validity Index (CGVI) was calculated by the mean total CVI, i.e., the sum of the CVI of each attribute divided by the number of attributes assessed^(24,25)^. This type of methodology is useful, especially when seeking to validate educational materials or interventions that seek to increase adherence to preventive practices or improve understanding about the use of diagnostic tests, such as HIV self-testing^(20,21)^. For instance, a study focused on assessing the content validity of an instrument on intention to use condoms among women in high-risk areas used the CVIG to ensure that the instrument’s items were clear and relevant^(22)^. In this study, CVIG calculation was performed by adding CVIs per item, and the mean was obtained (total CVI), with a minimum acceptance value of 0.80, determining the need for reformulation or exclusion of items that did not meet this criterion.

## RESULTS

### Educational booklet development

**Figure 1:**
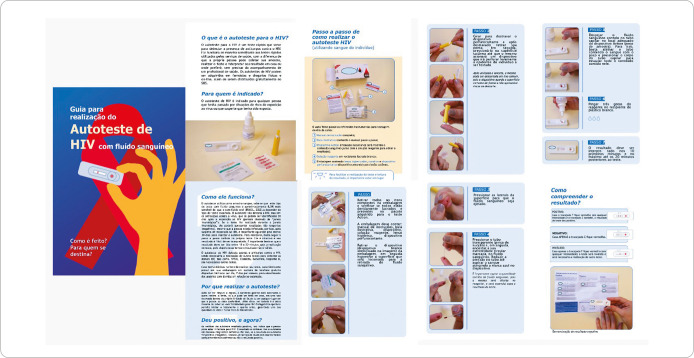
Educational booklet on HIV self-testing, Ribeirão Preto, São Paulo, Brazil, 2024

The educational booklet was created based on available scientific evidence and the researchers’ theoretical and practical experience on the topic. Information was organized in a question-and-answer format, accompanied by photographs illustrating how to perform an HIV self-test. This material was then submitted for layout and infographics (Figure 1).

The booklet is 12 pages long, written on A5 paper (148 x 210 mm), in color, and includes photographs and infographics. Its content is divided into two main sections: an educational introduction, consisting of six questions about HIV self-testing (What is HIV self-testing? Who is it for? How does it work? Why self-test? If you tested positive, what now? How do I understand the result?); and a practical guide with a detailed description of the kit’s materials and seven stages for performing a self-test with capillary blood, according to the manufacturer’s guidelines for the tests distributed by the SUS.

The step-by-step guide details self-testing stages, with illustrative photographs and specific instructions. The process ranges from cleaning the fingertips to interpreting the results (positive, negative, or invalid), with images and explanations that facilitate understanding. The booklet also provides guidance on the proper disposal of used materials. The aim was to use simple and direct language, avoiding technical jargon, and to include images of HIV self-test kit materials.

### Validity by experts

The educational booklet content and appearance validity was assessed by 13 experts, the majority of whom were female (8; 61.5%). Ten (76.9%) participants had nursing degrees and three (23.1%) had medical degrees, with seven (53.8%) originating from the Southeast region. Experts’ areas of expertise included six participants from teaching (46.2%), four from healthcare (30.8%), and two from research (23.1%).

The mean experience in the field was 13.3 years, with a standard deviation of 7.5 years. Eight participants (61.5%) worked in infectious diseases. In terms of training, three (25%) had postdoctoral degrees, four (33.3%) had doctoral degrees, three (25%) had master’s degrees, and two (16.7%) had specializations or residencies. Specialists reported having access to knowledge about HIV/AIDS in various ways, with six (46.2%) participants reporting having acquired it through practical experience in their workplaces, and two (15.4%) through publications in the field. Furthermore, nine (69.2%) specialists provided daily guidance on HIV self-testing.

The booklet’s relevance and general aspects obtained a total CVI of 0.92, ranging from 1 to 0.85. Furthermore, no suggestions for correction or improvement were made that impact the booklet quality or the validity result (Table 1).

**Table 1:** Assessment of the educational booklet relevance and general aspects by health experts (N=13), Ribeirão Preto, São Paulo, Brazil, 2024

Relevance and general aspects	TA	A	N	D	TD	CVI
Allows for understanding the use of self-test	10	2	1	-	-	0.92
Allows for understanding the effectiveness of self-test	10	2	1	-	-	0.92
Allows for understanding the benefits of self-test	8	3	1	1	-	0.85
Allows for understanding the eligibility criteria for using self-test	8	3	1	1	-	0.85
Allows for understanding the step-by-step process for performing self-test	11	2	-	-	-	1.00
The guidelines are applicable in practice	12	1	-	-	-	1.00
It is consistent with the needs of people who wish to perform a self-test	11	1	1	-	-	0.92
It can be used by healthcare professionals to encourage self-testing	12	-	-	-	1	0.92
The booklet clarifies questions about the topic covered	9	2	2	-	-	0.85
The booklet is relevant to the target audience	10	2	-	1	-	0.92
**Total CVI**						**0.92**

*CVI - Content Validity Index; TA - totally agree; A - agree; N - neutral; D - disagree; TD - totally disagree.*

The booklet’s content and language were assessed using 17 statements, focusing on aspects such as clarity, text sequence, relevance of topics, alignment with current literature, and use of technical terms. The CVI ranged from 1 to 0.85, resulting in a total CVI of 0.94. These results demonstrate robust booklet validity, highlighting its quality and effectiveness in conveying relevant information (Table 2).

**Table 2:** Assessment of the educational booklet content and written language by health experts (N=13), Ribeirão Preto, São Paulo, Brazil, 2024

Content and written language	TA	A	N	D	TD	CVI
The content provides all the necessary information for guidance on completing self-test	7	4	1	1	-	0.85
The content presented is clear	10	3	-	-	-	1.00
The content facilitates learning about self-test	12	1	-	-	-	1.00
The sequence of the booklet’s content is appropriate	12	1	-	-	-	1.00
The content allows for understanding of the topic	12	-	-	1	-	0.92
The content is in accordance with scientific literature	12	1	-	-	-	1.00
The guidelines were addressed correctly	12	1	-	-	-	1.00
The content is appropriate for the target audience	11	1	-	1	-	0.92
The guidelines are relevant to the target audience	12	-	-	-	1	0.92
The information is appropriate for the target audience	11	2	-	-	-	1.00
The information is presented in a context relevant to the target audience	11	1	-	-	1	0.92
The written language used in the booklet is appropriate	11	1	-	-	1	0.92
The written language is easy to understand	9	3	-	-	1	0.92
The technical terms are adequately defined	7	5	-	-	1	0.92
The concepts are addressed clearly	9	3	-	-	1	0.92
The concepts are addressed objectively	11	2	-	-	-	1.00
The booklet contains no errors or harmful ideas	12	-	-	-	1	0.92
**Total CVI**						**0.94**

*CVI - Content Validity Index; TA - totally agree; A - agree; N - neutral; D - disagree; TD - totally disagree.*

The final attribute assessed was the educational booklet aesthetic and visual quality, consisting of eight questions related to the assessment of the educational booklet aesthetic and visual quality. This assessment aimed to determine whether the material is attractive and arouses user interest through the appropriate use of colors and images, and whether the infographics created contribute to the retention of the described guidelines. This attribute obtained a total CVI of 0.98, reaching the minimum necessary level of agreement among experts (Table 3).

**Table 3:** Assessment of the educational booklet aesthetic and visual quality by health experts (N=13), Ribeirão Preto, São Paulo, Brazil, 2024

Aesthetic and visual quality	TA	A	N	D	TD	CVI
The booklet’s visual aspect is interesting	10	3	-	-	-	1.00
The booklet’s visual aspect is motivating for reading	10	3	-	-	-	1.00
The booklet has a satisfactory aesthetic/layout	10	3	-	-	-	1.00
The format and size of letters are satisfactory	10	1	2	-	-	0.85
The infographics created retain the written instructions	9	3	-	1	-	0.92
The infographics quality is satisfactory	9	4	-	-	-	1.00
The colors used do not interfere with reading	11	2	-	-	-	1.00
The layout facilitates understanding of the message	12	1	-	-	-	1.00
**Total CVI**						**0.98**

*CVI - Content Validity Index; TA - totally agree; A - agree; N - neutral; D - disagree; TD - totally disagree.*

The CVIG for validity with experts was calculated by dividing the mean total CVI of each attribute by the number of attributes (3), resulting in a result of 0.92, thus validating the educational booklet. Furthermore, no suggestions for corrections or improvements were made that would interfere with the booklet quality or the final validity result.

### Educational booklet validity by the target audience

The booklet semantic assessment included seven participants, mostly female (57.1%) and self-identified as brown (57.1%), aged between 25 and 46 years (mean of 35 years). Their education level ranged from elementary school (14.3%), high school (42.9%), and college (42.9%). The majority were single (42.9%), and all reported having sexual relations with men, with 42.9% having had relationships with partners living with HIV. Although 71.4% had received guidance on self-testing, 57.1% had never taken it.

In the target audience’s assessment, the total CVI was 0.95. Of the items addressed, 14 had a CVI higher than 0.8, while the two statements with a lower CVI were revised and did not refer to the booklet quality. It is important to emphasize that no suggestions for corrections or improvements were made (Table 4).

**Table 4:** Educational booklet assessment by the target audience (N=7), Ribeirão Preto, São Paulo, Brazil, 2024

Overall assessment	TA	A	N	D	TD	CVI
The information in the booklet is important	1	6	-	-	-	1
The information in the booklet helps to decide whether to take a self-test	4	3	-	-	-	1
I liked how the booklet’s content was presented	5	2	-	-	-	1
The images help to understand the booklet’s content	6	1	-	-	-	1
The colors used in the booklet are attractive	4	3	-	-	-	1
It helps to understand what a self-test is	6	1	-	-	-	1
It helps to understand the benefits of taking a self-test	5	2	-	-	-	1
It reflects the importance of taking a self-test	5	2	-	-	-	1
It helps to understand the possible limitations of a self-test	4	3	-	-	-	1
It helps to understand the precautions to take during a self-test	4	2	1	-	-	0.86
It helps to know where to get a self-test	4	2	1	-	-	0.86
It helps to understand the step-by-step process for taking a self-test	6	1	-	-	-	1
It makes it easier to find an HIV self-test	4	3	-	-	-	1
It helps to understand the indications for a self-test	2	1	-	3	1	0.43
It stimulates interest in taking a self-test	5	2	-	-	-	1
I know someone who would benefit from reading this booklet	3	2	-	2	-	0.71
**Total CVI**						**0.95**

*CVI - Content Validity Index; TA - totally agree; A - agree; N - neutral; D - disagree; TD - totally disagree.*

## DISCUSSION

In this study, an educational booklet on self-testing for HIV infection diagnosis through blood fluid was developed and validated in terms of content, appearance, and semantics. Its use is an important strategy for health literacy, contributing to promoting self-care and the autonomy to perform self-tests appropriately, safely, and confidentially. Booklets with appropriate guidelines help reduce errors, improve result interpretation, and expand access to diagnosis, especially for those facing barriers such as stigma or difficulties accessing healthcare services^(14,22)^.

HIV diagnosis was previously restricted to clinical settings, hindering access for part of the population. With self-testing, this access has expanded, allowing for autonomous and confidential testing. However, its effectiveness depends on knowledge about its appropriate use and overcoming fear and insecurity related to HIV diagnosis, highlighting the importance of clear information about HIV and prevention^(14,26,27)^.

HIV self-testing is an important innovation due to its widespread acceptance among young people and its ability to reach diverse social and demographic contexts. Despite the good implementation and interpretation of results by these groups, the inclusion of educational materials with clear, illustrated instructions can increase acceptance and correct use. Thus, combining self-testing with accessible guidance can increase population adherence and contribute to the achievement of global goals^(17,26)^.

Proper interpretation of self-test results is essential to guide the tested individual’s conduct and avoid doubts or misinterpretations. When properly understood, it can reduce the gap between taking the test and seeking follow-up care in cases of a positive result. Therefore, educational materials with accessible language and clear instructions are essential to guide the population on next stages, promoting timely referral, treatment adherence, and continuous care^(27)^.

Soft-hard technologies are essential for health promotion and prevention, as they facilitate education and dissemination of information, encouraging the adoption of self-care behaviors, such as serological testing^(28)^. Producing reliable material on HIV self-testing is crucial given the current state of information dissemination in digital environments. Rapid access to content on social media offers significant opportunities for health promotion, but it also poses challenges, particularly related to the reliability of the information shared. Misinformation can directly compromise individuals’ well-being, hindering access to fundamental rights such as health, education, and communication-the latter, in fact, is a strategic tool that mediates and strengthens other rights^(29)^.

In this context, the development of reliable digital materials on HIV self-testing emerges as a necessary response to ensure that the population has access to appropriate, clear, and scientifically based guidance. Health communication, when well-conducted, expands the reach of prevention actions, especially among groups seeking discreet and accessible alternatives to traditional healthcare services^(29)^.

Nurses’ and other professionals’ participation on the multidisciplinary team is essential in developing reliable educational materials, given their central role in providing guidance, support, and testing for HIV and other STIs. Booklet validity by experts in the field ensures that the content is appropriate, up-to-date, and evidence-based, preventing the dissemination of incorrect information. The booklet is expected to help reduce knowledge barriers, promote independent decision-making, expand access to frequent testing, and contribute to reducing HIV transmission, in line with global public health commitments^(30)^.

### Study limitations

A limitation of this study is the participation of users of a single service in the countryside of São Paulo, which may not fully reflect regional variations in language and comprehension. However, this limitation was mitigated by the collaboration of experts from different regions and by the use of clear and accessible language in the educational material, aiming to reach diverse audiences.

### Contributions to health

The HIV self-testing booklet is an accessible and low-cost educational tool that has the potential to support health and nursing teams’ work in promoting self-care, with a focus on HIV self-testing, contributing to reinforcing the role of nurses in providing health literacy and education, which favors expanded access to timely diagnosis of HIV infection.

## CONCLUSIONS

The educational booklet on HIV self-testing presented satisfactory CVIs, with values of 0.92 for content and 0.95 for content, appearance, and semantics. Its development prioritized clear and understandable language, including real images, infographics, and attractive graphic elements, aiming to facilitate understanding and reinforce guidelines for appropriate and timely self-testing. Future studies are recommended to assess its effectiveness in health literacy among different population segments, especially those in situations of greater social vulnerability, who tend to seek testing services less frequently. It is also expected that the booklet will be adapted to other formats and digital platforms, expanding its reach and applicability in various healthcare settings.

## Data Availability

The research data are available only upon request.
