# Supplementary material for: Self-test for HIV infection diagnosis: educational technology development and validity
Source: Rev Bras Enferm. 2026 Mar 30;79:e20240627. doi: 10.1590/0034-7167-2024-0627 (PMC13038221; doi:10.1590/0034-7167-2024-0627)
Supplement: Translation

# Translation: Autoteste para o diagnóstico da infecção pelo HIV: construção e validação de tecnologia educacional

**DOI:** 10.1590/0034-7167-2024-0627pt

**Authors:** Ana Luiza Carsoni Alves de Almeida, Karyanna Alves de Alencar Rocha, Júlia Freitas Gomes, Marcela Antonini, Henrique Ciabotti Elias, Elucir Gir, Renata Karina Reis

**Affiliations:** I Universidade de São Paulo. Ribeirão Preto, São Paulo, Brasil

**Keywords:** Autoteste, Diagnóstico da Infecção pelo HIV, Tecnologia Educacional, Estudo de Validação, Prevenção de Doenças.

## Abstract

**Objetivos:**

construir e validar cartilha educativa sobre o autoteste para diagnóstico do HIV.

**Métodos:**

desenvolvido em quatro etapas: revisão de literatura; elaboração de cartilha; validação por especialistas; e avaliação pelo público-alvo. O trabalho ocorreu entre fevereiro e julho de 2024. Para análise, foi utilizado o Índice de Validade de Conteúdo, considerando valores acima de 0,80 como válidos.

**Resultados:**

a cartilha, com 12 páginas, orienta sobre a realização e interpretação do autoteste de HIV. Foi validada por 13 especialistas e sete participantes, alcançando Índices de Validade de Conteúdo de 0,92 e 0,95, respectivamente. Os resultados indicam que o material apresenta validade de conteúdo, aparência e semântica.

**Conclusões:**

a cartilha “Guia completo para a realização do autoteste de HIV” tem potencial para fortalecer o letramento em saúde, apoiando atividades de aconselhamento e educação para a população na realização correta do autoteste.

## INTRODUÇÃO

A infecção pelo vírus da imunodeficiência humana (HIV) permanece um desafio significativo de saúde pública, mesmo após mais de quatro décadas de sua identificação. Em 2023, havia 39 milhões de pessoas vivendo com HIV no mundo, das quais 29,8 milhões estavam em tratamento com terapia antirretroviral (TARV); Ainda assim, globalmente foram registrados 1,3 milhão de novos casos e 630 mil mortes relacionadas à AIDS. Para enfrentar essa realidade, o Programa Conjunto das Nações Unidas sobre HIV/AIDS (UNAIDS) propôs uma ambiciosa meta global conhecida como 95-95-95, que propõe diagnosticar 95% das pessoas que vivem com HIV, tratar 95% dos diagnosticados e alcançar supressão viral em 95% dos tratados - um compromisso do qual o Brasil é signatário^(1)^. Países que avançaram na redução da incidência do HIV investiram em estratégias de prevenção combinada, enfrentando barreiras sociais, legais e estruturais, como estigma e desigualdade de gênero^(2)^. Entre essas estratégias, ampliar o acesso à testagem do HIV é fundamental, e informar a população sobre os locais de testagem é uma ação essencial nesse processo^(3)^.

Entre as estratégias promissoras para ampliar a prevenção e o diagnóstico do HIV, destaca-se a implementação do autoteste, recomendado desde 2016 pela Organização Mundial da Saúde e pelo Ministério da Saúde como parte da prevenção combinada^(3)^. Desta forma, ampliar a testagem da infecção pelo HIV é uma meta global e uma forma de oportunizar o diagnóstico da infecção pelo HIV e se constitui na etapa inicial para atingir os objetivos globais de controle da epidemia por meio do diagnóstico, tratamento e supressão viral. O autoteste apresenta vantagens como privacidade, conveniência e acessibilidade. Essa estratégia permite alcançar populações com baixos índices de testagem, inclusive aquelas que nunca realizaram testes para diagnóstico do HIV, contribuindo para o cumprimento das metas globais de diagnóstico^(4-6)^.

No Brasil, o autoteste é distribuído gratuitamente pelo Sistema Único de Saúde (SUS), que pode ser realizado pelo próprio usuário utilizando amostras de fluido oral ou sangue, promovendo a autonomia do usuário, sendo, inclusive, oferecido em teleatendimentos para ampliar sua distribuição e maior alcance de pessoas mais vulnerabilizadas que enfrentam dificuldades de acesso ao sistema de saúde^(7-9)^. Além disso, o autoteste tem sido utilizado como estratégia que contribui para prevenção combinada do HIV, especialmente na seleção de parceiros com base no resultado do teste, sendo considerado mais eficaz do que o relato verbal da condição sorológica das parcerias sexuais^(10)^.

Assim, destaca-se que a implementação do autoteste de HIV na Atenção Primária à Saúde configura-se como uma ferramenta eficaz para ampliar o acesso ao diagnóstico e superar barreiras enfrentadas por segmentos populacionais mais expostos a iniquidades sociais e mais vulnerabilizadas. Pela facilidade de uso e comodidade, permite que os indivíduos realizem o teste em locais de sua preferência, com resultados em 20 minutos^(8)^, promovendo o empoderamento dos indivíduos na gestão de sua saúde. Integrado a iniciativas de prevenção combinadas, como a Profilaxia Pré-Exposição (PrEP) e campanhas estratégicas, o autoteste facilita o diagnóstico oportuno e representa outra oportunidade para testes regulares de HIV. No entanto, para garantir a sua eficácia, é fundamental fornecer orientação e aconselhamento adequado sobre o uso do kit, além da simples entrega^(11-13)^.

O enfermeiro exerce papel fundamental juntamente com a equipe multiprofissional na prevenção da infecção pelo HIV e de outras infecções sexualmente transmissíveis (IST) na Rede de Atenção à Saúde do SUS^(14,15)^, atuando na realização do acolhimento pré e pós-testes e orientação dos usuários que realizam o autoteste para HIV, incluindo o encaminhamento para serviços especializados, gerenciamento de risco e início do tratamento em casos reagentes^(16)^. A testagem regular é uma estratégia essencial para o enfrentamento da epidemia. Recomenda-se que toda pessoa sexualmente ativa realize a testagem para HIV pelo menos uma vez ao ano e após qualquer situação de risco, como sexo sem preservativo, uso compartilhado de seringas ou violência sexual^(1-3)^. Porém, barreiras como estigma, discriminação e dificuldade de acesso aos serviços de saúde exigem a ampliação da divulgação do autoteste e orientações claras para sua apropriada utilização^(4,17,18)^.

O letramento em saúde é essencial para que as pessoas compreendam e utilizem informações sobre prevenção, testagem e tratamento do HIV. O autoteste amplia o acesso ao diagnóstico, especialmente entre populações em situação de vulnerabilidade^(19)^. Este estudo contribui com a educação em saúde por meio da construção e validação de uma cartilha com linguagem clara e acessível sobre o autoteste por fluido sanguíneo, com o objetivo de reduzir barreiras informacionais, promover autonomia, estimular a testagem regular e fortalecer as ações educativas, destacando o papel central do enfermeiro na prevenção do HIV e no cumprimento das metas globais de saúde.

## OBJETIVOS

Construir e validar o conteúdo e aparência de cartilha educativa sobre o autoteste para o diagnóstico da infecção pelo HIV.

## MÉTODOS

### Aspectos éticos

O estudo foi submetido e aprovado pelo Comitê de Ética em Pesquisa da instituição responsável, sob Parecer n^o^ 6.090.413, de 30 de maio de 2023. Seguiu as diretrizes e normas regulamentadoras da pesquisa com seres humanos no Brasil recomendadas pela Resolução nº 466/12 do Conselho Nacional de Saúde e pelo Ofício Circular nº 02/2021, que estabeleceu orientações para procedimentos em pesquisas realizadas em ambiente virtual. Todos os participantes foram esclarecidos sobre os objetivos, riscos e benefícios do estudo, e os dados foram coletados após concordância e assinatura do Termo de Consentimento Livre e Esclarecido (TCLE), obtido por meio de documentos físicos ou digitais.

### Desenho, período e local do estudo

Trata-se de estudo metodológico, desenvolvido em quatro etapas: revisão narrativa da literatura; construção de cartilha educativa; validação por especialistas; e avaliação pelo público-alvo. O estudo foi realizado em um Serviço de Atenção Especializada (SAE) localizado em um município de médio porte, no interior do estado de São Paulo, no período de dezembro de 2022 a julho de 2024. A equipe de pesquisa contou com pesquisadoras, enfermeiras e pós-graduandas que atuam na área de cuidados de enfermagem voltados a pessoas com IST.

### Etapas de desenvolvimento

A primeira etapa de elaboração de cartilha sobre o autoteste para HIV envolveu revisão narrativa da literatura, com ênfase nas recomendações do Ministério da Saúde sobre uso de autotestes por meio de fluido sanguíneo obtido pela coleta de sangue por punção digital disponibilizados pelo SUS^(9)^. Para ilustrar as etapas do autoteste em conjunto com o conteúdo explicativo, foram produzidas fotografias em alta resolução por um profissional realizadas no laboratório de prática clínica da Escola de Enfermagem de Ribeirão Preto da Universidade de São Paulo, com participação voluntária de uma integrante do grupo de pesquisa. As imagens foram captadas em ambiente adequado e utilizadas na composição de infográficos desenvolvidos por um *designer* especializado, que também foi responsável pela diagramação e apresentação visual da primeira versão da cartilha. O conteúdo selecionado foi organizado em formato de passo a passo em arquivo digital, com orientações relacionadas à testagem, prevenção e promoção da autonomia para realização do teste, integrando a descrição da realização do teste com conteúdo em educação em saúde.

Na segunda etapa, foi realizada validação de conteúdo e aparência a partir de um comitê de especialistas. A identificação e a seleção dos especialistas para este estudo foram fundamentadas em critérios que destacam a experiência e o conhecimento em saúde, adaptados do sistema de classificação de especialistas do Modelo de Validação de Fehring^(20)^. Esses critérios incluem ter experiência prática clínica recente de, no mínimo, um ano na área de interesse e/ou científica, ter concluído cursos de pós-graduação relacionados ao tema do estudo (mestrado e/ou doutorado) e ter publicação em revista indexada na temática de interesse.

Os especialistas foram selecionados por meio de busca na Plataforma *Lattes* e pelas técnicas de amostragem por bola de neve e por conveniência^(21)^, visando alcançar participantes acessíveis de diferentes regiões do país com experiência e atuação comprovadas, conforme os critérios de inclusão. O convite foi enviado por e-mail, contendo a apresentação do estudo, objetivos, justificativa para participação, além do *link* para o TCLE e para o questionário autoaplicável na plataforma *Research Electronic Data Capture* (REDCap^®^).

O questionário foi composto por duas partes: a primeira reunia informações sobre a identificação dos especialistas e sua experiência profissional; a segunda continha instruções para o preenchimento e os critérios de avaliação da cartilha, totalizando 35 questões. Dessas, dez estavam relacionadas à relevância e avaliação geral (compreensão das orientações, motivação e aplicabilidade prática); 17 abordavam a adequação do conteúdo e da linguagem escrita; e oito tratavam da qualidade estética e visual, com base em questionário proposto em outro estudo^(22)^. Os participantes tiveram a opção de fornecer sugestões e observações sobre os atributos avaliados, como o acréscimo de conteúdo relevante e identificação da necessidade de adequação de acordo com as evidências científicas.

Na terceira etapa, avaliação da cartilha educativa com o público-alvo, o objetivo era identificar evidência de qualidade, adequação de uso, confiabilidade e relevância, a fim de ser utilizado por um determinado segmento populacional. Assim, foi realizada avaliação semântica e de aparência da cartilha junto ao público-alvo^(23)^, composto por usuários atendidos no Centro de Especialidade Central, especificamente no SAE e Centro de Testagem e Aconselhamento (CTA) do município estudado.

Participaram do estudo adultos alfabetizados, com idade igual ou superior a 18 anos, soropositivos ou soronegativos para o HIV e que não tivessem realizado previamente o autoteste para HIV. A inclusão de usuários sem experiência anterior com autoteste teve como objetivo avaliar a compreensão da linguagem e das informações disponibilizadas na cartilha, sem a interferência de vieses relacionados ao conhecimento prévio. Foram excluídas da amostra pessoas com deficiências visuais ou cognitivas.

A seleção dos participantes ocorreu de forma consecutiva entre dezembro de 2023 e fevereiro de 2024. Após o recrutamento e assinatura do TCLE, os dados foram coletados individualmente em sala reservada, garantindo a confidencialidade, e registrados na plataforma REDCap^®^. Cada participante recebeu a cartilha impressa e respondeu a um questionário com questões sociodemográficas e 16 afirmações adaptadas de estudo anterior^(20)^, avaliando a adequação, relevância, clareza e pertinência do conteúdo, incluindo aspectos do autoteste de HIV e cuidados necessários para sua realização.

### Análise de dados

Para a análise de dados, realizou-se análise exploratória por meio de estatística descritiva utilizando medidas de frequência, tendência central e dispersão com o objetivo de descrever e caracterizar o perfil dos participantes.

Para análise da concordância entre os especialistas e público-alvo, utilizou-se o Índice de Validade de Conteúdo (IVC)^(24,25)^, o qual mensura a proporção de especialistas em concordância. As respostas a cada questão foram do tipo Likert com pontuação de 1 a 5: (1) discordo totalmente; (2) discordo; (3) neutro/indiferente; (4) concordo; e (5) concordo totalmente.

O cálculo do IVC de cada item foi realizado por meio de duas métricas. O Índice de Validade de Conteúdo por Item (IVC-I) foi calculado pela proporção de especialistas que consideraram um atributo relevante para “5” e “4” ou que necessitava de pequenas revisões, dividido pelo número total de especialistas. Os atributos que receberam pontuação “1”, “2” ou “3” foram revistos. Considerou-se neste estudo um IVC-I aceitável valor igual ou acima de 0,80, assegurando a validade e adequação dos atributos avaliados, conforme preconizado por diversos autores na área de avaliação de conteúdo^(23,24)^. O Índice de Validade de Conteúdo Global (IVCG) foi calculado pela média de IVC total, ou seja, a somatória dos IVC de cada atributo dividido pelo número de atributos avaliados^(24,25)^. Esse tipo de metodologia é útil, especialmente quando se busca validar materiais educativos ou intervenções que busquem aumentar a adesão a práticas preventivas ou melhorar o entendimento sobre o uso de testes de diagnóstico, como o autoteste de HIV^(20,21)^. Por exemplo, estudo focado em avaliar a validade de conteúdo de instrumento sobre a intenção de uso de preservativos entre mulheres em áreas de risco utilizou o IVCG para garantir que os itens do instrumento fossem claros e relevantes^(22)^. Neste estudo, o cálculo do IVCG foi realizado por meio da soma dos IVCs por item, e a média foi obtida (IVC total), com um valor mínimo de aceitação de 0,80, determinando a necessidade de reformulação ou exclusão de itens que não atendiam a esse critério.

## RESULTADOS

### Produção de cartilha educativa

A cartilha educativa foi construída com base em evidências científicas disponíveis e na experiência teórico-prática dos pesquisadores sobre o tema. As informações foram organizadas em formato de perguntas e respostas, acompanhadas de imagens fotográficas que ilustram a realização do autoteste para HIV. Esse material foi, então, encaminhado para diagramação e inclusão de infográficos (Figura 1).

**Figura 1:** Cartilha educativa sobre autoteste de HIV, Ribeirão Preto, São Paulo, Brasil, 2024

A cartilha possui 12 páginas, elaboradas em papel no formato A5 (148 x 210 mm), coloridas e com a inclusão de imagens fotográficas e infográficos. Seu conteúdo está dividido em duas partes principais: uma introdução didática, composta por seis questões sobre o autoteste de HIV (O que é o autoteste para o HIV? Para quem é indicado? Como ele funciona? Por que realizar o autoteste? Deu positivo, e agora? Como compreender o resultado?); e um guia prático com a descrição detalhada dos materiais que compõem o *kit* e os sete passos para a realização do autoteste com sangue capilar, conforme as orientações do fabricante dos testes distribuídos pelo SUS.

O passo a passo detalha as etapas do autoteste, com fotografias ilustrativas e orientações específicas. O processo abrange desde a higienização da polpa digital até a interpretação do resultado, podendo ser positivo, negativo e inválido, com imagens e explicação que favorecem a compreensão do texto. A cartilha também orienta sobre o descarte correto dos materiais utilizados. Buscou-se adotar linguagem simples e direta, evitando jargões técnicos, e incluir imagens dos materiais do kit de autoteste de HIV.

### Validação por especialistas

A avaliação da validade de conteúdo e aparência da cartilha educativa foi realizada por 13 especialistas, com a maioria sendo do sexo feminino (8; 61,5%). Entre os participantes, dez (76,9%) eram graduados em enfermagem e três (23,1%) em medicina, sendo que sete deles eram (53,8%) originários da região Sudeste. A área de atuação dos especialistas incluiu seis participantes da docência (46,2%), quatro da assistência (30,8%) e dois da pesquisa (23,1%).

O tempo médio de experiência na área foi de 13,3 anos, com um desvio padrão de 7,5 anos. Oito participantes (61,5%) trabalhavam na área da infectologia. Em termos de formação, três (25%) tinham pós-doutorado, quatro (33,3%), doutorado, três (25%), mestrado, e dois (16,7%), especialização ou residência. Os especialistas referiram ter contato com os saberes acerca do HIV/AIDS de diversas maneiras, sendo que seis (46,2%) participantes referiram tê-la conquistado pela experiência prática em seus locais de trabalho, e dois (15,4%), por publicações na área. Além disso, nove (69,2%) dos especialistas ofereciam orientações diárias sobre o autoteste de HIV.

A relevância e aspectos gerais da cartilha obtiveram um IVC total de 0,92, variando entre 1 e 0,85. Ademais, não foram feitas sugestões de correção ou melhoria que impactam a qualidade da cartilha ou o resultado da validação (Tabela 1).

**Tabela 1:** Avaliação da relevância e aspectos gerais da cartilha educativa pelos especialistas na área da saúde (N=13), Ribeirão Preto, São Paulo, Brasil, 2024

Relevância e aspectos gerais	CT	C	N	D	DT	IVC
Permite a compreensão do uso do autoteste	10	2	1	-	-	0,92
Permite a compreensão da eficácia do autoteste	10	2	1	-	-	0,92
Permite a compreensão dos benefícios do autoteste	8	3	1	1	-	0,85
Permite a compreensão dos critérios de elegibilidade para uso do autoteste	8	3	1	1	-	0,85
Permite a compreensão do passo a passo para realizar o autoteste	11	2	-	-	-	1,00
As orientações têm aplicabilidade na prática	12	1	-	-	-	1,00
É coerente com as necessidades das pessoas que desejam fazer um autoteste	11	1	1	-	-	0,92
Pode ser utilizada pelos profissionais de saúde para incentivar a realização do autoteste	12	-	-	-	1	0,92
A cartilha esclarece dúvidas sobre o tema abordado	9	2	2	-	-	0,85
A cartilha é relevante para o público-alvo	10	2	-	1	-	0,92
**IVC total**						**0,92**

*IVC - Índice de Validade de Conteúdo; CT - concordo totalmente; C - concordo; N - neutro; D - discordo; DT - discordo totalmente.*

A avaliação do conteúdo e da linguagem da cartilha foi realizada através de 17 afirmações, focando em aspectos como clareza, sequência do texto, relevância dos temas, alinhamento com a literatura atual e uso de termos técnicos. O IVC variou entre 1 e 0,85, alcançando um IVC total de 0,94. Esses resultados demonstram uma validação robusta da cartilha, evidenciando sua qualidade e eficácia na transmissão de informações relevantes (Tabela 2).

**Tabela 2:** Avaliação de conteúdo e linguagem escrita da cartilha educativa pelos especialistas na área da saúde (N=13), Ribeirão Preto, São Paulo, Brasil, 2024

Conteúdo e linguagem escrita	CT	C	N	D	DT	IVC
O conteúdo dispõe de todas as informações necessárias para orientação sobre a realização do autoteste	7	4	1	1	-	0,85
O conteúdo apresentado está claro	10	3	-	-	-	1,00
O conteúdo favorece a aprendizagem sobre o autoteste	12	1	-	-	-	1,00
A sequência do conteúdo da cartilha está adequada	12	1	-	-	-	1,00
O conteúdo permite a compreensão do tema	12	-	-	1	-	0,92
Os conteúdos estão de acordo com a literatura científica	12	1	-	-	-	1,00
As orientações foram abordadas corretamente	12	1	-	-	-	1,00
O conteúdo está adequado ao público-alvo	11	1	-	1	-	0,92
As orientações são pertinentes ao público-alvo	12	-	-	-	1	0,92
As informações são apropriadas ao público-alvo	11	2	-	-	-	1,00
As informações são apresentadas em um contexto pertinente ao público-alvo	11	1	-	-	1	0,92
A linguagem escrita utilizada na cartilha é adequada	11	1	-	-	1	0,92
A linguagem escrita é de fácil assimilação	9	3	-	-	1	0,92
Os termos técnicos estão adequadamente definidos	7	5	-	-	1	0,92
Os conceitos são abordados de forma clara	9	3	-	-	1	0,92
Os conceitos são abordados de forma objetiva	11	2	-	-	-	1,00
A cartilha não contém erro ou ideia prejudicial	12	-	-	-	1	0,92
**IVC total**						**0,94**

*IVC - Índice de Validade de Conteúdo; CT - concordo totalmente; C - concordo; N - neutro; D - discordo; DT - discordo totalmente.*

O último atributo avaliado foi a qualidade estética e visual da cartilha educativa, composta por oito questões relacionadas à avaliação da qualidade estética e visual da cartilha educativa. O objetivo desta avaliação era determinar se o material é atrativo e desperta o interesse dos usuários por meio do uso adequado de cores e imagens, e se os infográficos elaborados contribuem para a retenção das orientações descritas. Esse atributo obteve IVC total de 0,98, alcançando o valor mínimo necessário de concordância entre os especialistas (Tabela 3).

**Tabela 3:** Avaliação da qualidade estética e visual da cartilha educativa pelos especialistas na área da saúde (N=13), Ribeirão Preto, São Paulo, Brasil, 2024

Qualidade estética e visual	CT	C	N	D	DT	IVC
O aspecto visual da cartilha é interessante	10	3	-	-	-	1,00
O aspecto visual da cartilha é motivador para a leitura	10	3	-	-	-	1,00
A cartilha apresenta uma estética/layout satisfatório	10	3	-	-	-	1,00
O formato e tamanho das letras estão satisfatórios	10	1	2	-	-	0,85
Os infográficos elaborados retêm as orientações por escrito	9	3	-	1	-	0,92
A qualidade dos infográficos é satisfatória	9	4	-	-	-	1,00
As cores utilizadas não atrapalham a leitura	11	2	-	-	-	1,00
A diagramação favorece o entendimento da mensagem	12	1	-	-	-	1,00
**IVC total**						**0,98**

*IVC - Índice de Validade de Conteúdo; CT - concordo totalmente; C - concordo; N - neutro; D - discordo; DT - discordo totalmente.*

O IVCG da validação com os especialistas foi realizado pela média de IVC total de cada atributo dividido pelo número de atributos (3), cujo resultado foi 0,92, validando, dessa forma, a cartilha educativa. Ademais, não foram feitas sugestões de correção ou melhoria que interferissem na qualidade da cartilha ou no resultado final da validação.

### Validação de cartilha educativa pelo público-alvo

A avaliação semântica da cartilha contou com sete participantes, majoritariamente do sexo biológico feminino (57,1%) e autodeclarados pardos (57,1%), com idades entre 25 e 46 anos (média de 35 anos). A escolaridade variou entre ensino fundamental completo (14,3%), médio completo (42,9%) e superior completo (42,9%). A maioria era solteira (42,9%,) e todos relataram ter relações sexuais com homens, sendo que 42,9% já haviam se relacionado com parceiros vivendo com HIV. Embora 71,4% tenham recebido orientações sobre o autoteste, 57,1% nunca o haviam realizado.

Na avaliação pelo público-alvo, o IVC total foi de 0,95. Dos itens abordados, 14 apresentaram IVC superior a 0,8, enquanto as duas afirmações com IVC inferior foram revistas e não se referiam à qualidade da cartilha. Importante ressaltar que não foram feitas sugestões de correção ou melhoria (Tabela 4).

**Tabela 4:** Avaliação da cartilha educativa pelo público-alvo (N=7), Ribeirão Preto, São Paulo, Brasil, 2024

Avaliação geral	CT	C	N	D	DT	IVC
As informações na cartilha são importantes	1	6	-	-	-	1
As informações da cartilha ajudam a decidir sobre a realização do autoteste	4	3	-	-	-	1
Eu gostei de como o conteúdo da cartilha foi apresentado	5	2	-	-	-	1
As imagens ajudam a entender o conteúdo da cartilha	6	1	-	-	-	1
As cores utilizadas na cartilha são atrativas	4	3	-	-	-	1
Possibilita compreender o que é o autoteste	6	1	-	-	-	1
Possibilita compreender os benefícios em fazer um autoteste	5	2	-	-	-	1
Reflete a importância em fazer um autoteste	5	2	-	-	-	1
Possibilita a compreensão de possíveis limitações do autoteste	4	3	-	-	-	1
Possibilita a compreensão dos cuidados durante o autoteste	4	2	1	-	-	0,86
Permite saber onde buscar um autoteste	4	2	1	-	-	0,86
Permite compreender o passo a passo para realização autoteste	6	1	-	-	-	1
Facilita a busca pelo autoteste de HIV	4	3	-	-	-	1
Permite a compreensão da indicação para o autoteste	2	1	-	3	1	0,43
Despertam o interesse na realização do autoteste	5	2	-	-	-	1
Eu conheço alguém que se beneficiaria lendo essa cartilha	3	2	-	2	-	0,71
**IVC total**						**0,95**

*IVC - Índice de Validade de Conteúdo; CT - concordo totalmente; C - concordo; N - neutro; D - discordo; DT - discordo totalmente.*

## DISCUSSÃO

No presente estudo, foi construída e validada quanto ao conteúdo, aparência e semântica uma cartilha educativa sobre o autoteste para o diagnóstico da infecção pelo HIV por meio do fluido sanguíneo. O seu uso é uma estratégia importante para o letramento em saúde, que contribui promovendo o autocuidado e a autonomia para realização dos autotestes de forma apropriada segura e confidencial. Cartilhas com orientações adequadas ajudam a reduzir erros, melhorar a interpretação dos resultados e ampliar o acesso ao diagnóstico, especialmente para quem enfrenta barreiras como estigma ou dificuldades de acesso aos serviços de saúde^(14,22)^.

O diagnóstico do HIV antes era restrito a ambientes clínicos, dificultando o acesso de parte da população. Com o autoteste, esse acesso foi ampliado, permitindo testagem autônoma e sigilosa. Porém, sua efetividade depende do conhecimento sobre o uso apropriado e da superação do medo e insegurança relacionados ao diagnóstico da infecção pelo HIV, destacando a importância de informações claras sobre o HIV e prevenção^(14,26,27)^.

O autoteste de HIV é uma inovação importante por sua ampla aceitação entre jovens e sua capacidade de alcançar diversos contextos sociais e demográficos. Apesar da boa execução e interpretação dos resultados por esses grupos, a inclusão de materiais educativos com instruções claras e ilustradas pode aumentar a aceitação e o uso correto. Assim, a combinação do autoteste com orientações acessíveis pode ampliar a adesão populacional e contribuir para o alcance das metas globais^(17,26)^.

A interpretação adequada dos resultados do autoteste é essencial para orientar a conduta da pessoa testada e evitar dúvidas ou interpretações equivocadas. Quando bem compreendida, pode reduzir a lacuna entre a realização do teste e a busca por acompanhamento no serviço de saúde em casos de resultado reagente. Assim, materiais educacionais com linguagem acessível e instruções claras são fundamentais para orientar a população sobre os próximos passos, promovendo o encaminhamento oportuno, adesão ao tratamento e cuidado contínuo^(27)^.

As tecnologias leve-duras são essenciais para a promoção e prevenção em saúde, pois facilitam a educação e disseminação de informações incentivando a adoção de comportamentos de autocuidado, como a testagem sorológica^(28)^. A produção de um material fidedigno sobre o autoteste de HIV se mostra fundamental, diante do atual cenário de disseminação de informações em ambientes digitais. O acesso rápido a conteúdo nas mídias sociais oferece oportunidades significativas para promoção da saúde, mas também impõe desafios, especialmente relacionados à confiabilidade das informações compartilhadas. A desinformação pode comprometer diretamente o bem-estar dos indivíduos, dificultando o acesso a direitos fundamentais como saúde, educação e comunicação - esta última, inclusive, é uma ferramenta estratégica que intermedeia e fortalece os demais direitos^(29)^.

Nesse contexto, a elaboração de materiais digitais confiáveis sobre o autoteste de HIV surge como uma resposta necessária para garantir que a população tenha acesso a orientações apropriadas, claras e cientificamente embasadas. A comunicação em saúde, quando bem conduzida, amplia o alcance das ações de prevenção, especialmente entre grupos que buscam alternativas discretas e acessíveis ao serviço tradicional de saúde^(29)^.

A participação de enfermeiros e demais profissionais da equipe multiprofissional é essencial na elaboração de materiais educativos confiáveis, devido ao seu papel central na orientação, acolhimento e realização da testagem de HIV e outras ISTs. A validação da cartilha por especialistas na temática garante que o conteúdo seja adequado, atualizado e baseado em evidências, prevenindo a disseminação de informações incorretas. Espera-se que a cartilha contribua para reduzir barreiras de conhecimento, promover decisões autônomas, ampliar o acesso à testagem frequente e colaborar para redução da transmissão do HIV, em alinhamento com os compromissos globais de saúde pública^(30)^.

### Limitações do estudo

Como limitação do estudo, destaca-se a participação de usuários de um único serviço do interior paulista, o que pode não refletir plenamente as variações regionais de linguagem e compreensão. No entanto, essa limitação foi atenuada com a colaboração de especialistas de diferentes regiões e com o uso de linguagem clara e acessível no material educativo, visando atingir públicos diversos.

### Contribuições para a área da saúde

A cartilha sobre o autoteste de HIV é uma ferramenta educativa acessível e de baixo custo que tem potencial para apoiar a atuação da equipe de saúde e enfermagem na promoção do autocuidado, com foco na realização do autoteste de HIV, contribuindo para reforçar o papel do enfermeiro na realização do letramento e educação em saúde, que favorecem a ampliação do acesso ao diagnóstico oportuno da infecção pelo HIV.

## CONCLUSÕES

A cartilha educativa sobre o autoteste para o HIV apresentou IVCs satisfatórios, com valores de 0,92, para conteúdo, e 0,95, para conteúdo, aparência e semântica. Sua elaboração priorizou linguagem clara e compreensível, com inclusão de imagens reais, infográficos e elementos gráficos atrativos, visando facilitar a compreensão e reforçar as orientações para a realização apropriada e oportuna do autoteste. Recomenda-se que estudos futuros avaliem sua efetividade no letramento em saúde entre diferentes segmentos populacionais, especialmente aqueles em situação de maior vulnerabilidade social, que tendem a buscar menos os serviços de testagem. Espera-se, ainda, que a cartilha seja adaptada para outros formatos e plataformas digitais, ampliando seu alcance e aplicabilidade em diversos contextos assistenciais.

## Data Availability

Os dados de pesquisa só estão disponíveis mediante solicitação.
